# Whole Reproductive System Non-Negative Matrix Factorization Mass Spectrometry Imaging of an Early-Stage Ovarian Cancer Mouse Model

**DOI:** 10.1371/journal.pone.0154837

**Published:** 2016-05-09

**Authors:** Martin R. L. Paine, Jaeyeon Kim, Rachel V. Bennett, R. Mitchell Parry, David A. Gaul, May D. Wang, Martin M. Matzuk, Facundo M. Fernández

**Affiliations:** 1 School of Chemistry and Biochemistry, Georgia Institute of Technology, Atlanta, GA, 30332, United States of America; 2 Department of Pathology & Immunology, Baylor College of Medicine, Houston, TX, 77030, United States of America; 3 Department of Computer Science, Appalachian State University, Boone, NC, 28608, United States of America; 4 School of Biology, Georgia Institute of Technology, Atlanta, GA, 30332, United States of America; 5 Integrated Cancer Research Center, Georgia Institute of Technology, Atlanta, GA, 30332, United States of America; 6 Walter H. Coulter Department of Biomedical Engineering Georgia Institute of Technology, Atlanta, GA, 30332, United States of America; 7 Department of Molecular and Cellular Biology, Baylor College of Medicine, Houston, TX, 77030, United States of America; 8 Department of Molecular and Human Genetics, Baylor College of Medicine, Houston, TX, 77030, United States of America; 9 Department of Pharmacology, Baylor College of Medicine, Houston, TX, 77030, United States of America; 10 Center for Drug Discovery, Baylor College of Medicine, Houston, TX, 77030, United States of America; 11 Center for Reproductive Medicine, Baylor College of Medicine, Houston, TX, 77030, United States of America; 12 Institute of Bioengineering and Biosciences, Georgia Institute of Technology, Atlanta, GA, 30332, United States of America; West China Second Hospital, Sichuan University, CHINA

## Abstract

High-grade serous carcinoma (HGSC) is the most common and deadliest form of ovarian cancer. Yet it is largely asymptomatic in its initial stages. Studying the origin and early progression of this disease is thus critical in identifying markers for early detection and screening purposes. Tissue-based mass spectrometry imaging (MSI) can be employed as an unbiased way of examining localized metabolic changes between healthy and cancerous tissue directly, at the onset of disease. In this study, we describe MSI results from *Dicer-Pten* double-knockout (DKO) mice, a mouse model faithfully reproducing the clinical nature of human HGSC. By using non-negative matrix factorization (NMF) for the unsupervised analysis of desorption electrospray ionization (DESI) datasets, tissue regions are segregated based on spectral components in an unbiased manner, with alterations related to HGSC highlighted. Results obtained by combining NMF with DESI-MSI revealed several metabolic species elevated in the tumor tissue and/or surrounding blood-filled cyst including ceramides, sphingomyelins, bilirubin, cholesterol sulfate, and various lysophospholipids. Multiple metabolites identified within the imaging study were also detected at altered levels within serum in a previous metabolomic study of the same mouse model. As an example workflow, features identified in this study were used to build an oPLS-DA model capable of discriminating between DKO mice with early-stage tumors and controls with up to 88% accuracy.

## Introduction

High-grade serous carcinoma (HGSC), a subtype of ovarian cancer also known as high-grade serous ovarian cancer, is responsible for 70% of all ovarian cancer deaths and yet the origin and early progression of this deadly disease remains poorly understood.[1–4] Reliable screening tests in clinical practice are currently unavailable, and the asymptomatic course through early stages of the disease make early detection of HGSC extremely difficult. Consequently, most HGSC cases (> 95%), and by extension the majority of ovarian cancer cases, are diagnosed at advanced stages leading to poor 5-year survival rates–approximately 44.6% for all cases diagnosed during 2004–2010.[1, 5–7] However, when the cancer is diagnosed early and is confined to the primary tumor site, the 5-year survival rate increases to over 90%.[8] It is therefore imperative that early detection methods are developed to combat ovarian cancer mortality.

The identification of biochemical changes within tissues from early- to late-stage HGSC could provide a means for selecting markers that could be monitored in blood or urine for early detection and diagnosis. A major challenge associated with this approach, however, is difficulty in finding patients with early-stage ovarian cancer, as early stages are asymptomatic. Recent mouse models of ovarian cancer, with HGSC originating in the fallopian tube or ovary, present a unique opportunity to overcome this challenge.[9–11] When two critical genes are conditionally disabled (*Dicer*^flox/flox^ *Pten*^flox/flox^ *Amhr2*^cre/+^), these double-knockout (DKO) mice develop fallopian tube-originated HGSCs, which then spread to the ovary before metastasizing throughout the abdominal cavity, causing ascites and leading to death.[9] Alternatively, double-mutant mice carrying mutant p53 and *Pten* deletion produce ovary-originated HGSCs as well as non-HGSCs in the ovary with normal fallopian tubes. [10] In addition, triple-mutant mice, (*p53*^LSL-R172H/+^ *Dicer*^flox/flox^ *Pten*^flox/flox^ *Amhr2*^cre/+^) that include mutant p53, the most frequently observed mutations in human HGSC (~97%), also develop fallopian tube-originated HGSC with the identical pattern of metastasis as DKO mice. Though both DKO and TKO models duplicate equally well the clinical nature of human HGSC, the DKO model has also been characterized at the molecular level, with DKO HGSC exhibiting molecular similarity to human HGSC. Therefore, in this study DKO mice were selected for further investigation using mass-spectrometry-based imaging analyses.

In mass spectrometry imaging (MSI), mass spectra are recorded at discrete spatial points across the tissue sample, leading to a datacube with a mass spectrum for each (x,y) pixel. Two-dimensional false-color images that map the spatial distribution of specific analytes are created from these data.[12] Two well-established MSI methods that have been applied to cancer studies are matrix-assisted laser desorption/ionization (MALDI),[13–18] and desorption electrospray ionization (DESI).[19–25] Specifically for ovarian cancer, several MALDI MSI studies have been published focusing on both protein and peptide biomarker identification.[26–29] Only a report by Liu et al has investigated the potential role of metabolites using MSI.[30] In this study, MALDI experiments were directed by transcriptomic analysis and focused specifically on the detection of sulfatides, a subclass of glycosphingolipids. DESI MSI, on the other hand, has only been applied to the study of the molecular-level changes observed between pre- and post-ovulation, but not to early cancer biomarker detection.[31]

Given the vast amount of chemical information generated in MSI, extracting the most biologically-relevant information related to a specific factor may not be straightforward, requiring visual inspection and comparison of hundreds to even thousands of images for each experiment. To simplify MSI data analysis, comparison of spectra from multiple regions or multiple samples is possible using region-of-interest (ROI) selection.[32] ROI comparisons are straightforward, averaging spectra obtained at each pixel from a selected region, and are best suited for analyses where histological boundaries are well defined. However, in cases where histological boundaries are less clear or when new biological hypothesis are being generated, manually defining ROI’s can be challenging. In the investigation of disease progression in large tissue sections, for example, chemical markers may be sparsely distributed and at low abundances, making their detection by ROI selection difficult.

Semi-supervised methods employing clustering algorithms that automatically assign segmentation maps according to patterns of co-localized molecules can be employed in such scenarios. However, these methods still divide the data into discrete spatial segments and require the user to inspect many individual ion images to confirm they match with the corresponding segment.[33] Unsupervised methods based on statistical algorithms are therefore preferred for data mining of large, heterogeneous tissue samples, requiring minimal human intervention.[34, 35] Such algorithms have been investigated for analysis of MSI datasets including Principal Component Analysis (PCA)[36], Maximum Autocorrelation Factorization[37], K-Means[38] and Fuzzy C-Means[39] clustering, Probabilistic Latent Semantic Analysis[40], and Non-Negative Matrix Factorization (NMF)[41]. Comparison of the Pearson correlation of output component images against true sample images showed that of all these methods, NMF had the highest mean image correlation.[35] Also, because of the built-in non-negativity constraints, NMF component images are easily interpreted and better suited for mass spectrometric data than other multivariate statistical methods, such as PCA.[42, 43]

Herein we follow up on our serum metabolomics study of the *Dicer-Pten* DKO mice with a DESI MSI study of the complete reproductive system of the same mouse model.[44] DESI MSI multivariate data analysis was carried out with a new version of omniSpect[45], now capable of handling larger (~TB) MSI datasets. By separating the MSI data into multiple NMF components via omniSpect, features representing metabolomic changes between healthy and cancerous tissue were readily visualized. The corresponding NMF “component spectra” describing these images were then studied, identifying chemical species altered in the HGSC tumor microenvironment, several of which had also been previously detected at the serum level.[44]

## Materials and Methods

### Dicer-Pten Double-Knockout (Dicer-Pten DKO) Mice

*Dicer-Pten* DKO (*Dicer*^flox/flox^ *Pten*^flox/flox^ *Amhr2*^cre/+^) mice were generated by mating males (*Dicer*^flox/flox^ *Pten*^flox/flox^ *Amhr2*^cre/+^) with females (*Dicer*^flox/flox^ *Pten*^flox/flox^). Female *Dicer*^flox/flox^ *Pten*^flox/flox^ (a genotype not carrying *Amhr2*^cre/+^) mice were used as controls. All mice were obtained from in-house breeding at the Baylor College of Medicine and were housed in a vivarium with a controlled temperature of 21°C. They were fed 5053 irradiated PicoLab Rodent Diet 20 and had access to drinking water supplied in bottles. *Dicer*^flox/flox^ *Pten*^flox/flox^ *Amhr2*^cre/+^ DKO mice used in this study develop ascites as tumors metastasize. Once mice begun to show signs of ascites, they were monitored daily. Mice were recommended for euthanasia when the ascites volume reached 10% of the animal's body weight. All mice used in this study (10 in total) were sacrificed before reaching the limit for euthanasia via an overdose inhalation of carbon dioxide to prevent unnecessary pain and distress in accordance to the animal protocol (AN716) approved by the Institutional Animal Care and Use Committee (IACUC) at the Baylor College of Medicine.

### Sample Preparation

The whole reproductive system of DKO mice (including uterus, fallopian tubes, and ovaries) were excised and flash frozen at -80°C. The intact excised tissue was embedded in an aqueous solution of gelatin (10% w/v) and carboxymethyl cellulose (5% w/v) and frozen at -80°C. Embedded tissue blocks were placed in the chamber of a Cryostar NX70 cryostat (Thermo Scientific Inc., San Jose, CA, USA) for 30 min and then sectioned at 20 μm thickness with both the cutting block and sample block maintained at -20°C. Tissue sections were thaw mounted onto Superfrost Plus Micro Slides (VWR International LLC., Radnor, PA, USA) and kept at -80°C until analyzed.

### Desorption Electrospray Ionization-Mass Spectrometry Imaging

DESI-MS images were acquired using a custom-built ion source consisting of an Omni Spray DESI sprayer (Prosolia Inc., Indianapolis, IN) capable of fine adjustments using manual stages and multi-axis platforms (Thorlabs, Inc., Newton, NJ). The following DESI-MS geometric and experimental variables were optimized for the imaging experiment: height of sprayer-tip above surface (5 mm), incident angle (55°), sprayer-tip distance from MS inlet (15 mm), inlet height above surface (<1 mm), spray solvent (methanol), solvent flow rate (5 μL min^-1^), nebulizing gas (nitrogen), nebulizing gas pressure (100 psi), and spray voltage (-5 kV). The mass spectrometer inlet used was specifically designed for DESI-MS imaging and protrudes 18 cm from the front of the instrument, allowing access to larger sample surfaces.

While the DESI sprayer and MS inlet were held stationary, the sample to be imaged was moved in a “comb” shaped pathway, allowing the DESI sprayer to raster the entire sample surface in 2-dimensions using an OptiScan II motorized microscope stage (Prior Scientific Inc., Rockland, MA) controlled by a Labview VI program described previously.[19, 45] The stage velocity was set to 160 μm s^-1^ in the *x*-dimension with a line step of 200 μm in the *y*-dimension. Negative-ion mass spectra were acquired on an Exactive Plus Orbitrap mass spectrometer (Thermo Scientific, San Jose, CA) over the range of *m/z* 400–1000 in centroid mode after optimizing the following experimental variables: mass resolution (17,500 at *m/z* 200), automatic gain control target (1e^6^ ions), microscans per scan (2), s-lens voltage (75 V), maximum injection time (100 ms), and capillary temperature (250°C).

### Data Processing

The MS imaging data file as a single, continuous acquisition, along with Labview time and output position files were uploaded to the omniSpect web-based server (http://cs.appstate.edu/omnispect).[45] From the Labview-generated stage position and timing information, omniSpect creates an image cube line-by-line using mass spectra collected on the first pass along the comb-shaped path. The original mass spectra are linearly interpolated onto a regularly spaced grid along the x-axis.[46]

For collected spectra, omniSpect first estimates a full profile using a common logarithmically-spaced *m/z* scale for all scans and applying a small Gaussian window around each peak. We used a standard deviation of *m/z* 0.001 and a bin-width of *m/*z 0.0005 at *m/z* 850 for these data. In general, reducing the bin-width allows the Gaussian center to more accurately represent the centroid data peak center (*i*.*e*., the experimental *m/z* value) with surrounding bins being filled to accommodate mass shifts over the course of the experiment. The standard deviation therefore corresponds to the tolerated variance of this mass shift and represents the expected peak width if the data were collected in profile mode. The precision for centroided data is configurable in the latest version of omniSpect (http://cs.appstate.edu/omnispect/) with three preset settings: *m/z* ± 0.1, 0.01, and 0.001. At the highest precision, this approach generates data cubes with very large dimensions but very sparse data. For example, typical mouse MSI data produced an image cube of size [805×59×2556741] which would require 904 GB if stored as double precision values. Because only 0.06% of the elements are nonzero, using a sparse matrix representation results in files requiring less than 2 GB in memory.

Given an MSI data cube, NMF models each component spectrum as the non-negative linear combination of source spectra and each component ion image as the non-negative linear combination of source images. For example, source spectra in a given tissue sample might pertain to different ions combinations or “components”, being produced at various tissue types, each occupying different but possibly correlated spatial distributions. NMF recovers the non-negative component spectra and spatial distributions by minimizing the difference between the modeled and the observed data.

First, the 3-D data cube is converted into a 2-D matrix by vectorizing the pixel coordinates into a single dimension. The user selects the number of components to extract and NMF estimates them. Among the many algorithms for NMF, we use the Alternating Least Squares (ALS) approach to minimizing the Frobenius norm[47]:

$$D=\frac{1}{2}{{\left\|V-\mathrm{WH}\right\|}^{2}}_{F}$$

where **V** represents the data matrix, **W** represents the component images, and **H** represents the component mass spectra. Each column of **W** and corresponding row of **H** represents the spatial distribution and mass spectra from one component, respectively. Their product, **WH**, represents the linear combination of components that approximates the data. The algorithm starts by initializing **H** with positive values. Then, it solves for the **W** that minimizes *D* for fixed **H** using the least squares solution:

$$W={\mathrm{VH}}^{T}{\left({\mathrm{HH}}^{T}\right)}^{\#}$$

where # represents the Moore-Penrose pseudoinverse. Any negative values in **W** are replaced with zeros and the process is repeated for **H**. Specifically, *D* is minimized for the new value of **W**:

$$H=({W^{T}W)}^{\#}W^{T}V$$

Again, negative values are replaced with zeros, and the process is repeated, alternating between **W** and **H** until convergence. After estimation, component images are represented as 2-dimensional false-color plots and the relative abundance of ions within the spectra are normalized to the base-peak within the set of NMF components.[47]

### Data Analysis

Mass spectral features from the fourth NMF component were cross-referenced with the extracted and curated list of spectral features obtained following UPLC−MS analysis of serum from 14 early-stage tumor (ET) DKO mice and 11 controls.[44] UPLC-MS spectral features were extracted from the data using MZmine 2.0 software[48] and involved chromatogram alignment, peak identification and integration, peak area extraction, and normalization after curation of the data matrix. Curation of the data consisted of the removal of signals that were present in the blank samples, the solvent, or were not present in at least 50% of the serum samples. Mass spectral features overlapping between the selected NMF component and the curated UPLC-MS list were used to build a model for sample class discrimination via orthogonal partial least squares discriminant analysis (oPLS-DA) using MATLAB (MATLAB version 7.13.0, The MathWorks, Natick, MA with PLS_Toolbox v.6.71, Eigenvector Research, Wenatchee, WA).

## Results

**Fig 1:**
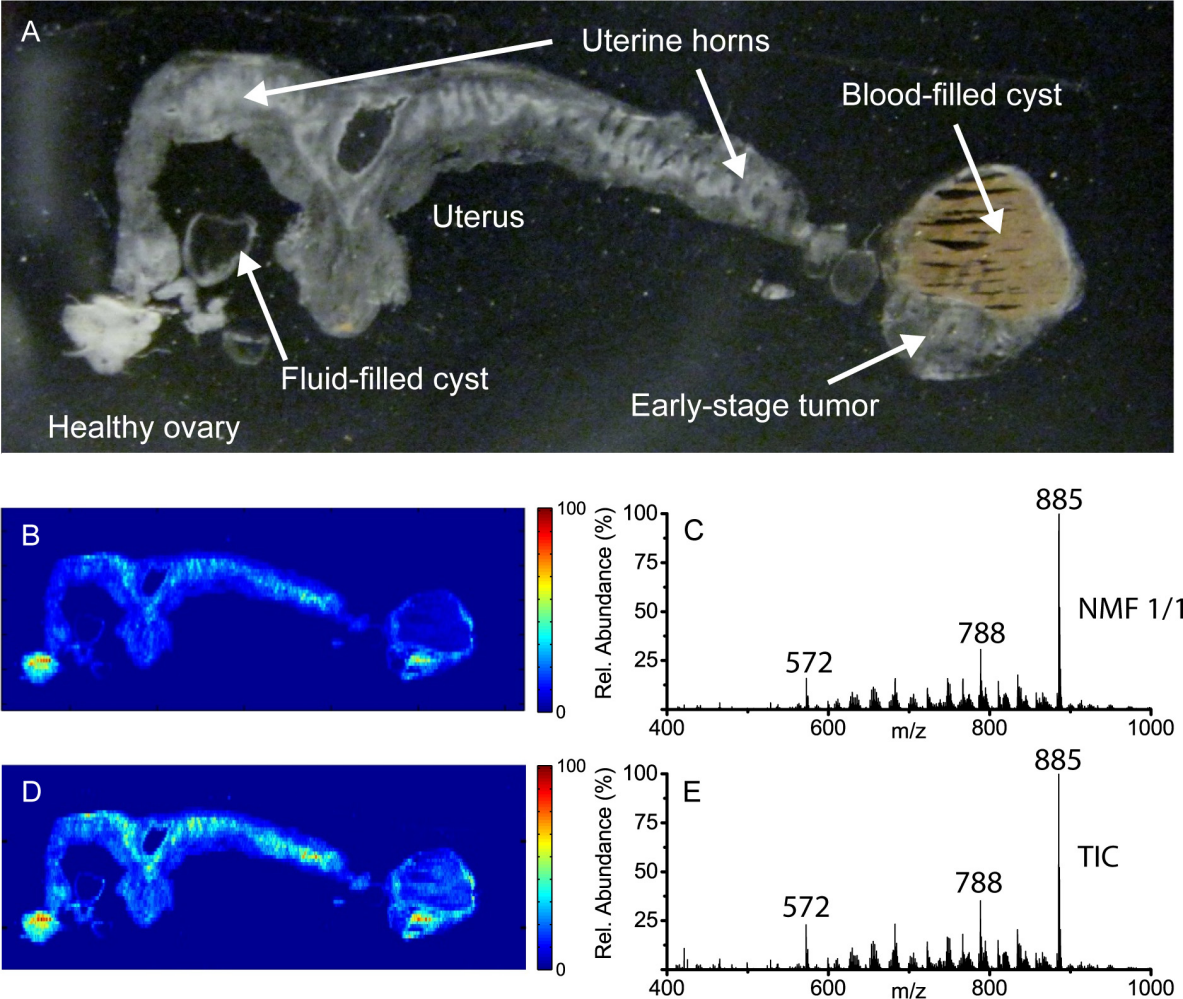
(a) Photograph of a thin tissue section of a DKO mouse reproductive system thaw-mounted onto a glass slide. (b) A single component NMF image representing the DESI-MS negative-ion mode data for the DKO mouse reproductive system and (c) the corresponding NMF spectrum. (d) The total ion current (TIC) image for the DESI-MS negative-ion mode data and (e) the corresponding TIC spectrum.

Four *Dicer-Pten* DKO mice at age 8 months were sacrificed and the intact reproductive organs were collected. One mouse displayed a large primary tumor with a blood-filled cyst at the end of one uterine horn and a healthy ovary with a pre- or non-cancerous cyst at the other uterine horn (Fig 1A). This sample was selected for MSI analysis as it was hypothesized that by imaging the healthy ovary, pre- or non-cancerous cyst, and mature tumor together, direct spectral comparisons could be made between the different tissue regions. Differing spectral components detected, particularly those localized within the tumor/cyst region, could then be characterized and assessed for their biological role or as potential markers of disease.

A negative-ion mode DESI-MS image was acquired from a 20 μM thick section of the complete reproductive system excised from the aforementioned DKO mouse. Fig 1 shows an optical image of the tissue sample along with the false-color image and corresponding spectra for both the total ion chronogram (TIC) and single-component NMF result. Comparison of both images and spectra illustrate how closely the ALS algorithm can approximate the data cube factors and therefore how well NMF results represent the true data. The images presented in Fig 1B and 1D are similar but not identical, as NMF approximates every spectrum in this image as a scaled version of one component spectrum. Regions of the NMF component image that do not match the TIC image indicate those regions have spectra that differ from the TIC spectrum. For example, the tumor/cyst region on the right shows a higher intensity on the false-color scale in the TIC image but the average spectrum for that region is not modeled well by the ‘average’ spectrum for the entire image. Therefore, by comparison, the tumor/cyst region is represented with lower relative intensity on the false-color scale in the single-component NMF image. However, the spectra in Fig 1C and 1E are almost identical with peak lists and relative abundances conserved. Progressively segmenting the data into two or more components using NMF could therefore provide meaningful results regarding biological differences related to disease progression by revealing these spectral differences. Determining the optimum number of NMF components so as to model true biological variance while rejecting unwanted noise, technical variation, and artifacts is thus central to understanding the level of useful information contained in these data.

**Fig 2:**
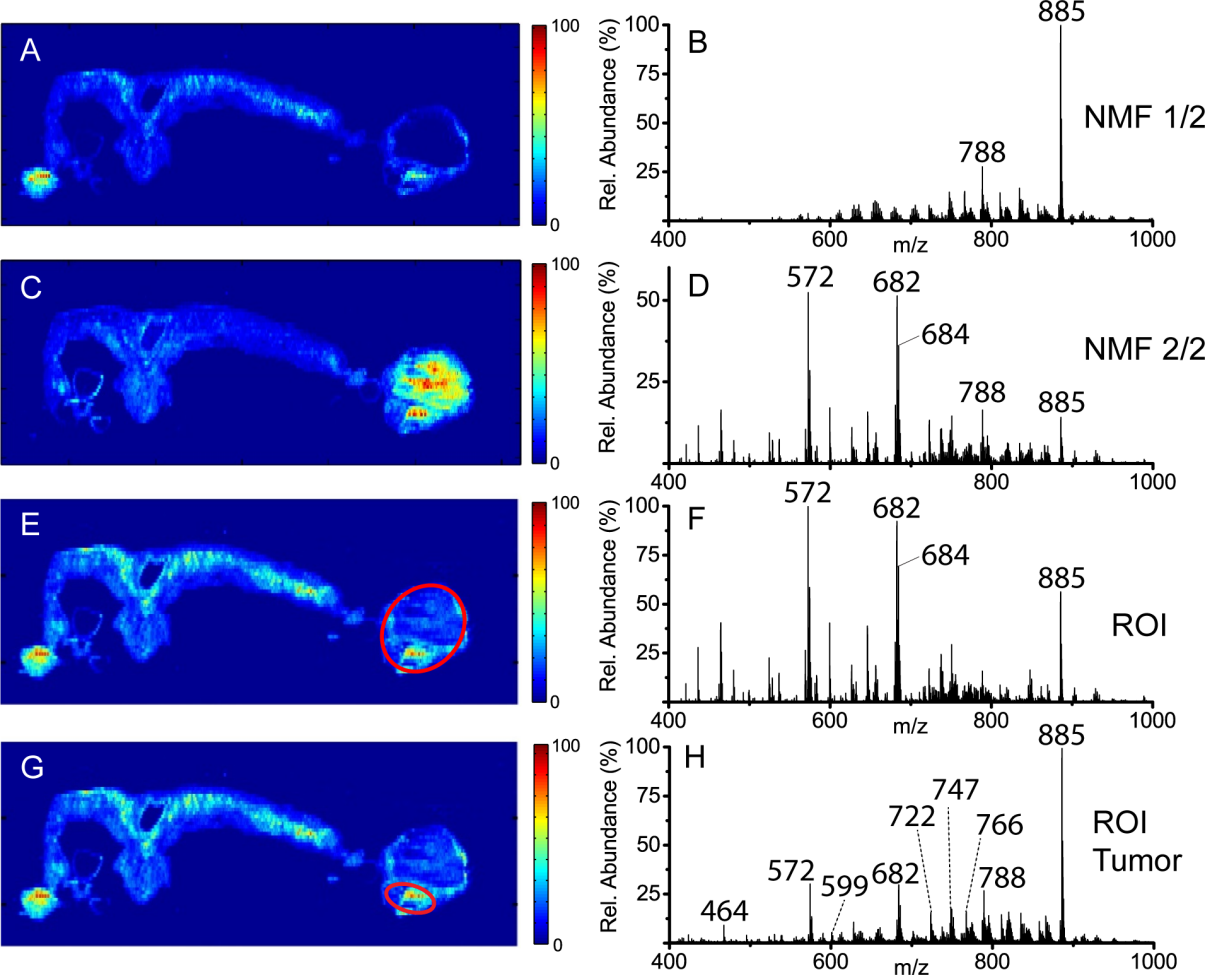
The resulting NMF component images (a) and (c), and corresponding spectra (b) and (d) from splitting the DESI-MS negative-ion mode acquisition into two NMF components. (e) The TIC image from the same dataset showing the region-of-interest (ROI) manually selected for the early-stage tumor and blood-filled cyst areas (red oval) and (f) the corresponding averaged spectrum from the ROI. (g) The TIC image showing the region-of-interest (ROI) selected for the early-stage tumor site only (red oval) and (f) the corresponding averaged spectrum from the ROI.

Fig 2A and 2C show the results from splitting the data cube into two NMF components. The image in Fig 2A resembles that of Fig 1B with the base peak at *m/z* 885.5436 dominating the corresponding spectra for both images. The accurate mass of this peak suggests it corresponds to the [M-H]^-^ ion of a phosphatidylinositol (PI) with 38 total carbons and 4 total double bonds in the acyl chains (theo. [M-H]^-^; *m/z* 885.5499). This lipid species is one of the most abundant acidic lipid species present in mammalian cell membranes and is routinely detected when performing MSI of mammalian tissues.[49] The most notable difference between Figs 2A and 1B is the reduced signal intensity in Fig 2A within the tumor/cyst region. Fig 2C shows the second NMF component and reveals a stark contrast to the first NMF component, with increased signal intensities in the tumor/cyst region and decreased intensities in the healthy ovary region. Fig 2D represents the corresponding spectrum for the image in Fig 2C, revealing the species that contribute to the increased signal intensity within the tumor/cyst region. Three major species were observed in Fig 2D at *m/z* 572.483, 682.589, and 684.595. For the species at *m/z* 572.483 and 684.595, an M+2 peak was observed at a relative abundance ratio of 3:1, suggesting the presence of one chlorine atom within each species. For *m/z* 682.589, the M+2 signal overlaps with the species at *m/z* 684.595. The species at *m/z* 684.595 has a relative abundance much higher than that expected for the ^37^Cl isotope of *m/z* 682.589. Therefore, ions at *m/z* 684.595 are attributed to a separate chemical species with possible contribution from a ^37^Cl isotope. Interestingly, these three ions were also detected in a study that investigated cellular lipid extracts from murine tumor cell lines by ESI-MS and were identified as the [M+Cl]^-^ ions of C16, C24:1, and C24:0 ceramides (theo. *m/z* 572.4815, *m/z* 682.5911, and *m/z* 684.6067, respectively).[50] Elevation of these three metabolites have been reported to be involved in tumor-induced dendritic cell apoptosis by down-regulating the phosphoinositide-3-kinase (PI3K) pathway.[50] By releasing these immunosuppressive signaling compounds, the tumor creates a favorable microenvironment whereby it can evade the host’s immune response and proliferate.[51]

Region-of-interest (ROI) spectra from the combined early-stage tumor and blood-filled cyst region (Fig 2E) and the early-stage tumor by itself (Fig 2G) were extracted from the data cube using a MATLAB script for comparison to the corresponding NMF component image. By manually selecting an ROI for a specific tissue region, an averaged spectrum is generated from spectra for each pixel within the selected region. The second NMF component spectrum (Fig 2D) and the ROI spectrum (Fig 2F) share a high degree of similarity, with all of the major ions detected in the ROI spectrum also present in the NMF component spectrum, albeit with slight differences in relative abundances. The major differences observed between Fig 2D and 2F arise due to NMF placing greater emphasis on combinations of species detected with higher degrees of variation from other regions of the image. To confirm that the major peaks identified in Fig 2D and 2F are actually present in the tumor region and are not simply associated with the biological differences between blood and solid tissue, an ROI spectrum of the early-stage tumor without the blood-filled cyst is shown in Fig 2H. The corresponding ROI spectrum contains all the peaks present above 5% relative abundance within Fig 2D. However, in Fig 2H these same peaks are present at much lower relative abundances due to the highly abundant membrane lipids, *i*.*e*., PI(38:4) at *m/z* 885.54. The ROI method provides a more accurate representation of the all species present in the selected region; however, NMF can isolate species that distinguish between regions, potentially aiding in the discovery of species with relatively low abundances that might be altered. This attribute is critical in trying to identify disease progression through tissue without supervised user intervention to define boundaries between diseased and healthy tissue.

**Fig 3:**
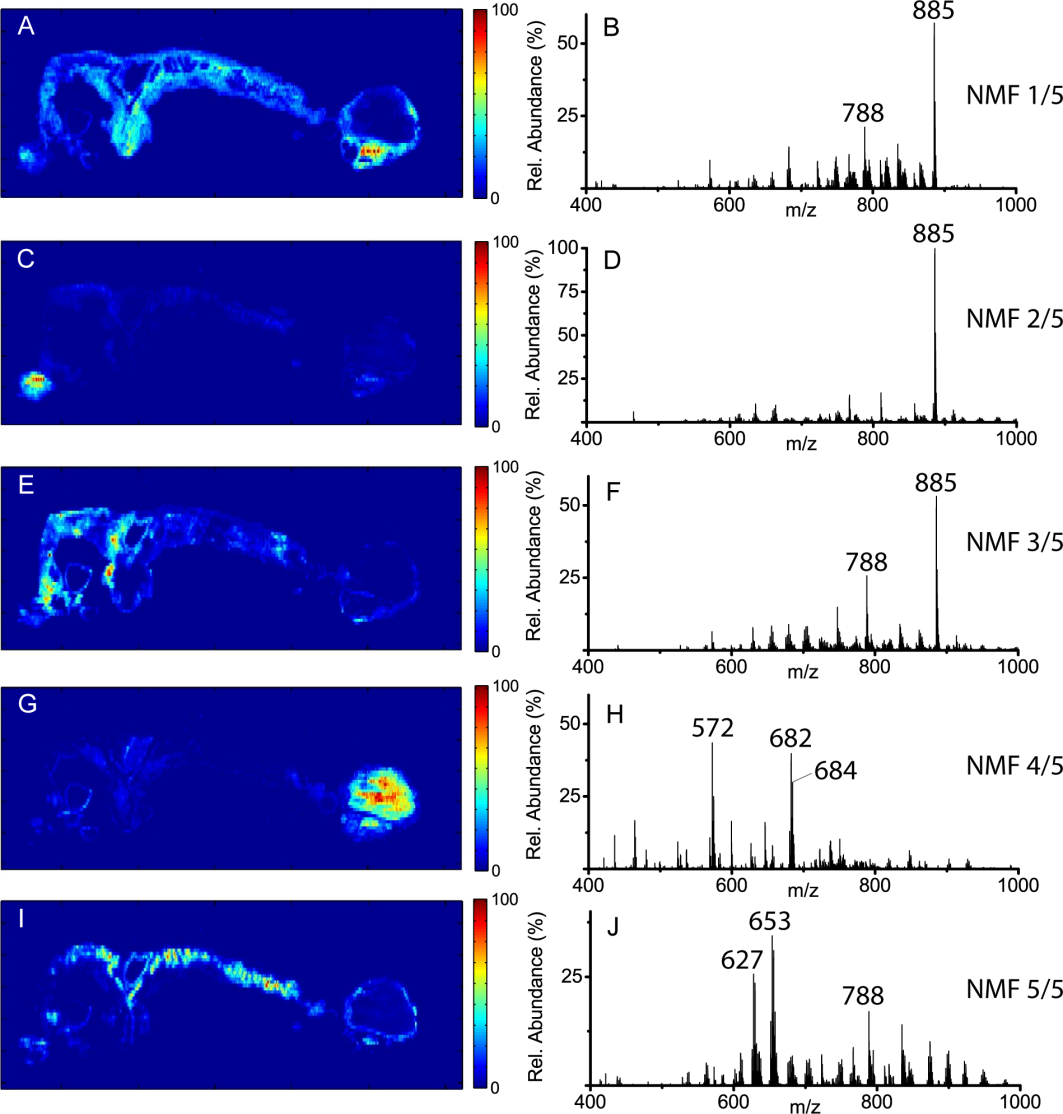
The resulting NMF component images and corresponding component spectra from splitting the DESI-MS negative-ion mode dataset into five NMF components.

Splitting the dataset into three, four, and five NMF components (S1 and S2 Figs, and Fig 3, respectively) progressively segregates the signals that are distinctly associated with the tumor/cyst region from the rest of the healthy tissue. What emerges is an image primarily representing the healthy ovary, one or more images (depending on the number of components selected) exhibiting different ion distributions throughout the uterus and healthy ovary, and an image showing elevated species in the tumor/cyst region. The spectra corresponding to the healthy ovary component image (Fig 3C) that acts as our pseudo-control sample for this experiment is dominated by the ion at *m/z* 885.5436 with all other ions observed at a relative abundance of 5% or less. The progressive NMF factorization of the signals contributing to the uterus (Fig 3A, 3E and 3I) show a lower relative abundance of the ion at *m/z* 885.5436 than Fig 3C. In addition, a greater relative contribution from other ions in these spectra is observed, particularly those in the lipid region between *m/z* 600–1000 from potential phosphatidylethanolamines (PE; *m/z* 716.5247, 722.5070, 746.5053, 766.5324, and 794.5395), phosphatidylserines (PS; *m/z* 788.5369, 810.5229, 834.5229), phosphatidylglycerols (PG; *m/z* 747.5108), and phosphatidylinositols (PI; *m/z* 833.5120, 857.5111, 909.5489).[49]

Of the five NMF component images, Fig 3A exhibits the highest intensity in the tumor region. However, Fig 3A also shows a relatively high correlation to the NMF component spectra (Fig 3B) throughout the majority of the tissue section. Although the peaks in Fig 3B are elevated in the tumor region, they are not necessarily ideal biomarkers to differentiate between tumor and healthy tissues. This is supported by the majority of peaks identified in Fig 3B being common mammalian cell membrane lipids that are known to be elevated due to an increased rate of lipid synthesis in cancerous tissues.[52] On the contrary, Fig 3G shows a much greater relative difference between the tumor/cyst region and the remainder of the tissue section. It is true that the highest intensities and the majority of the most intense pixels are observed in the cyst but there is still a significant number of pixels with elevated intensities in the tumor region. As the component image in Fig 3G almost exclusively shows the tumor/cyst region with minimal contributions from other tissue areas, this component should be highly representative of metabolic changes within the tumor microenvironment. The three remaining NMF components describe presumably non-cancerous regions of the tissue (*i*.*e*., healthy ovary and majority of the uterus). Therefore, the component image in Fig 3G is expected to contain minimal contributions from chemical species not specific to the cancer biology, with the corresponding spectrum shown in Fig 3H.

The performance of NMF as an unsupervised segmentation algorithm applied to MSI data was assessed by the comparison with a more typical clustering algorithm such as K-Means clustering. The K-Means results from segmenting the MSI dataset into *k* = 5 clusters is shown in S3 Fig. Four of the five K-Means clusters were spatially and spectrally very similar to the NMF results observed in Fig 3, with the blood-filled cyst, healthy ovary, and remaining tissue all segmented into separate clusters. For the K-Means analysis, one of the clusters contained large contributions from the chemical background signal and therefore that cluster correlated spatially with the region outside the biological tissue. Segmentation of this background region was not observed within the NMF results, instead the uterus was separated into three segments (compared to two with the K-Means clustering). It is evident that NMF is more suitable for MSI data analysis as NMF provides the degree of correlation of every pixel to the spectral approximation set for each component, whereas K-Means only gives a binary indication for each pixels inclusion to that specific cluster. Thus NMF images provide a more informative descriptor for MSI data than K-Means clustering.

To illustrate the selective power of NMF, ions detected within the tumor spectrum at an abundance of ≥ 5% relative to the base peak are listed in Table 1. Tentative structural identities of these ions are suggested based on accurate mass measurements, database searches, and literature reporting the detection of similar species in murine or human carcinomas.[53, 54] Eighteen ions representing fifteen different metabolites were tentatively identified in Table 1, including six ceramide species, two sphingomyelin species, various lysophospholipids, cholesterol sulfate, and bilirubin. Not surprisingly, sphingolipids comprise the majority of metabolites in this list, as they are known to play a significant biological role in cell cycle progression, telomerase function, and cell migration.[55] In addition, cholesterol sulfate is known for its stabilizing and regulatory role as a cell membrane component in a range of mammalian tissues and has also been detected in elevated levels in human uterine cervical carcinoma, squamous cell cancers, and prostate cancer tissues.[56, 57] The alteration in levels of bilirubin, a heme degradation product, may stem from the host’s immune response to the tumor. It has been reported that bilirubin plays a role in the defense against cancer by interfering with pro-cancerogenic signaling pathways.[58] Interestingly, our previous serum metabolomics study of the same *Dicer-Pten* DKO mouse model also identified bilirubin as a marker for detecting early-stage HGSC, highlighting the potential for MSI to complement more traditional biomarker discovery studies.

**Table 1:** Major ions detected from NMF components that exhibit elevated relative abundance at the primary tumor site and blood- and their tentative structural identification based on accurate mass measurements.

Monoisotopic mass (exp.)	Elemental formula	Monoisotopic mass (theo.)	Rel. error (ppm)	Molecular species	Tentative structural identification
**436.2826**^†^	C_21_H_43_NO_6_P	436.2834	1.8	[M-H]^-^	LPE(P-16:0)
**464.3139**	C_23_H_47_NO_6_P	464.3147	1.7	[M-H]^-^	LPE(O-18:1), LPE(P-18:0)
**465.3054**^†^	C_27_H_46_O_4_S	465.3044	1.3	[M-H]^-^	Cholesterol Sulfate
**480.3091**^†^	C_23_H_47_NO_7_P	480.3096	0.8	[M-H]^-^	LPE(18:0)
**524.2975**^†^	C_24_H_47_NO_9_P	524.2994	3.6	[M-H]^-^	LPS(18:0)
**536.5044**^†^	C_34_H_66_NO_3_	536.5048	0.9	[M-H]^-^	Cer(d34:1)
**572.4827**	C_34_H_67_NO_3_Cl	572.4815	2.1	[M+Cl]^-^	Cer(d34:1)
**583.2567**^†^	C_33_H_36_N_4_O_6_	583.2562	0.3	[M-H]^-^	Bilirubin^‡^
**599.3209**^†^	C_27_H_52_O_12_P	599.3202	1.2	[M-H]^-^	LPI(18:0)
**626.5328**	C_38_H_73_NO_3_Cl	626.5285	6.8	[M+Cl]^-^	Cer(d38:2)
**646.6124**	C_42_H_80_NO_3_	646.6144	3.1	[M-H]^-^	Cer(d42:2)
**648.6272**	C_42_H_82_NO_3_	648.6300	5.9	[M-H]^-^	Cer(d42:1)
**656.5800**	C_40_H_79_NO_3_Cl	656.5754	7.0	[M+Cl]^-^	Cer(d40:1)
**658.5919**	C_40_H_81_NO_3_Cl	658.5910	1.5	[M+Cl]^-^	Cer(d40:0)
**682.5891**	C_42_H_81_NO_3_Cl	682.5911	2.9	[M+Cl]^-^	Cer(d42:2)
**684.5952**	C_42_H_83_NO_3_Cl	684.6061	15.9	[M+Cl]^-^	Cer(d42:1)
**737.5318**^†^	C_39_H_79_N_2_O_6_P	737.5369	6.9	[M+Cl]^-^	SM(d34:1)
**847.6430**	C_47_H_93_N_2_O_6_PCl	847.6465	4.1	[M+Cl]^-^	SM(d42:2)

^†^Mass also detected in LC-MS analysis of serum from the same *Dicer-Pten* DKO mouse model diagnosed with early-stage high-grade serous ovarian cancer.[44]

^‡^Metabolite identified in serum and reported as a discriminatory marker between control mice and *Dicer-Pten* DKO mouse model diagnosed with early-stage high-grade serous ovarian cancer.[44]

**Fig 4:**
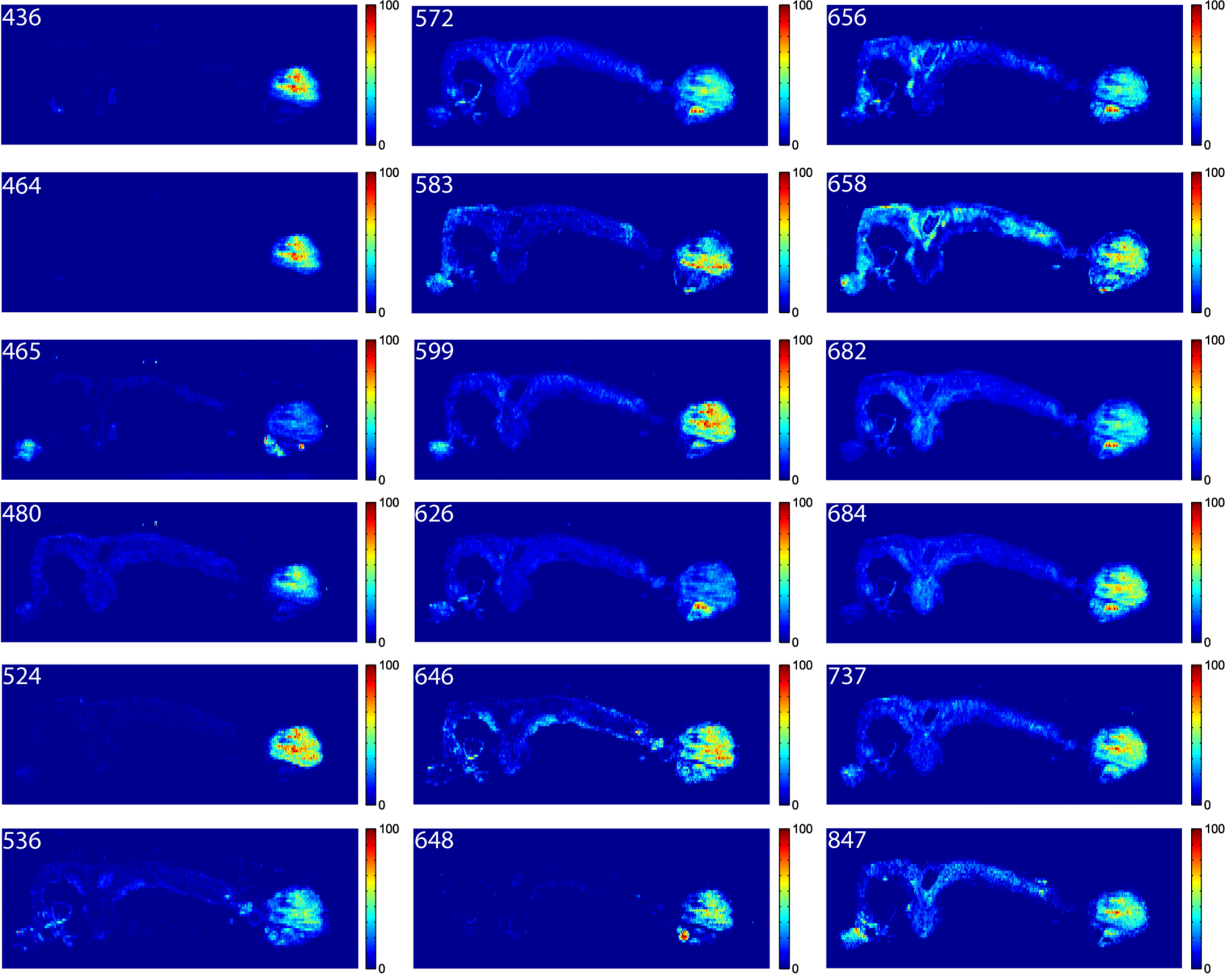
Extracted ion images shown as 2-dimensional false-color plots for the eighteen *m/z* values listed in Table 1 (see above).

To validate the NMF MSI results, individual images for each *m/z* value reported in Table 1 were extracted. These are shown in Fig 4 as false-color plots. All eighteen extracted ion images indicated elevated ion abundances within the tumor/cyst region. A few species also displayed a unique distribution throughout the remaining tissue that may be indicative of their normal biological role, or suggest their involvement in the formation of pre-cancerous lesions. For example, the extracted ion image for *m/z* 465 tentatively identified as the [M-H]^-^ ion for cholesterol sulfate shows elevated abundances in both the tumor/cyst region and the healthy ovary, but not the uterus. This observation is consistent with previous studies that suggest cholesterol sulfate is heavily utilized in the mitochondria of rodent ovaries as a substrate for the synthesis of sulfated steroids.[56] Since cholesterol is a precursor for steroid hormones, which are primarily synthesized in the ovary, this may also suggest that the ovaries, though not necessarily the origin of the cancer in the DKO mouse model, may play an indirect role in carcinogenesis via steroid hormones.

The metabolites listed in Table 1 were also cross-referenced against the list of serum metabolites observed *via* UPLC−MS analysis of the same mouse model.[44] In that study, blood samples were collected from 23 *Dicer-Pten* DKO mice (*Dicer*^*flox/flox*^ *Pten*^*flox/flox*^ *Amhr2*^*cre/+*^) containing early-stage tumors and compared to 21 control mice (*Dicer*^*flox/flox*^ *Pten*^*flox/flox*^). UPLC−ESI-MS analysis of the blood samples was performed using a Waters ACQUITY UPLC H Class system fitted with a Waters ACQUITY UPLC BEH C8 column (2.1 × 100 mm, 1.7 μm particle size) and coupled to a Xevo G2 QTOF mass spectrometer. The chromatographic method for sample analysis involved elution with water (mobile phase A) and methanol (mobile phase B) at a flow rate of 0.40 mL min^−1^ using the following gradient program: 0−15 min 20−90% B; 15−19 min 90% B. Due to similarities in ionization mechanism, DESI-MS yields mass spectra similar to those obtained by ESI-MS and with methanol being the major solvent for both studies, detection of the same classes of compounds from both blood and tissue is expected.[59]

**Fig 5:**
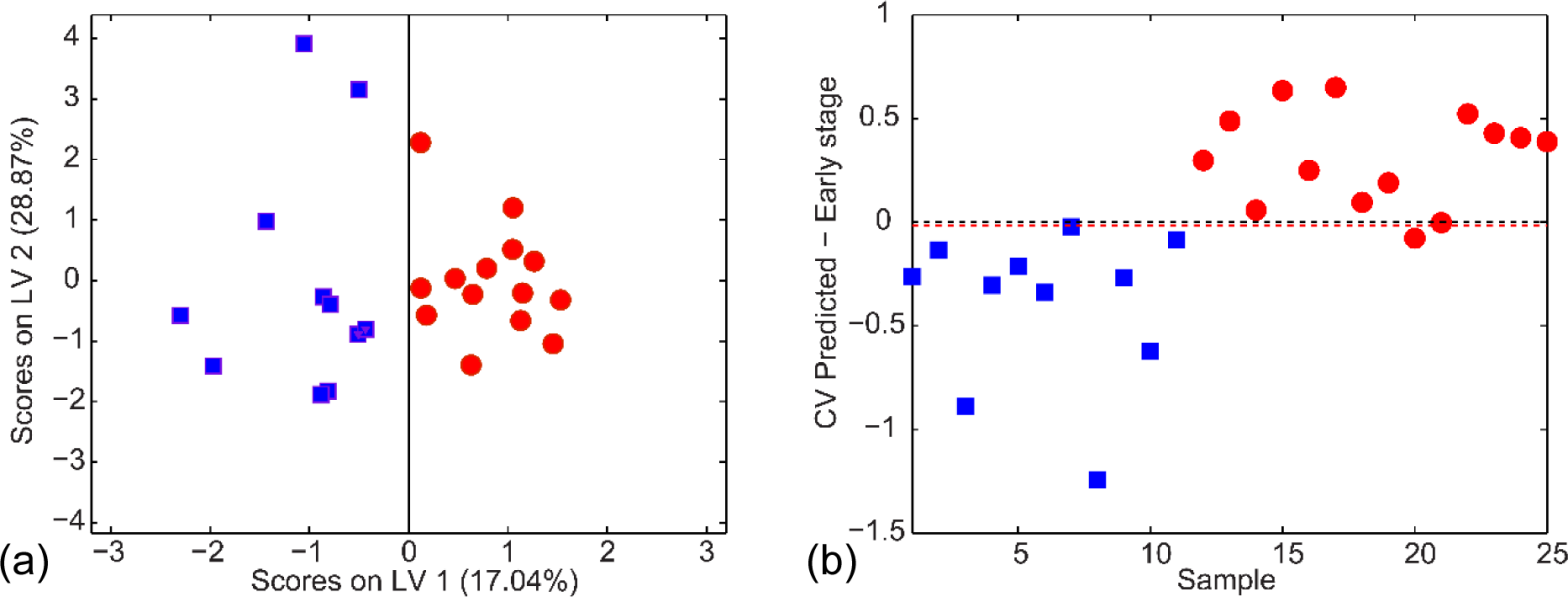
(a) oPLS-DA calibration scores plot using the 8 discriminant metabolites overlapping between the fourth DESI MSI NMF component and those detected in serum via UPLC-MS. The model consisted of 2 LVs with 17.04% and 22.87% total captured X- and Y-block variances, respectively. The performance characteristics of the oPLS-DA analysis were 88% accuracy, 91% sensitivity, and 86% specificity. (b) Corresponding ET cross-validated prediction plot for panel (a). There were only 2 misclassified mice using leave-one-out cross-validation.

Of the eighteen features listed in Table 1, eight were also present in the curated list of UPLC-MS-detected species (denoted with †). These eight metabolites were used to build a model for sample class discrimination between control and ET DKO mice via oPLS-DA. Performance characteristics of the oPLS-DA analysis that included the eight selected metabolic features (Fig 5A and 5B) were 88%, 91%, and 86% for the cross-validated accuracy, sensitivity, and specificity, respectively. A total of 2 mice samples, both ET DKO samples, were misclassified. This 2 latent variable model interpreted 17.04% and 22.87% variance from the X- (feature peak areas) and Y-block (mouse class membership), respectively.

Interestingly, of the eight metabolites used for oPLS-DA, six of those were measured in lower abundance in the ET DKO mice sera relative to the control samples. This lower serum abundance may be indicative of the elevated cellular metabolism occurring within the tumor microenvironment in order to support its growth and survival.[60] These metabolites may be biologically necessary for tumor proliferation and sequestered by the tumor cells from circulating blood, thereby decreasing their abundance relative to control blood samples. Conversely, metabolite depletion in the blood may also be the result of increased uptake in the tumor area due to the host’s immune response, which could also explain the changes in serum observed for bilirubin. Additionally, two metabolites identified by MSI and detected at elevated levels in DKO mice sera relative to control mice were Cer(d34:1) at *m/z* 536.5044 and lysophosphatidylinositol (18:0) (LPI(18:0)) at *m/z* 599.3209. This specific LPI, among several other LPI species, have been detected at increased levels in both plasma and ascites fluid of ovarian cancer patients and has been implicated as a signaling molecule in various cellular systems.[61–63]

**Fig 6:**
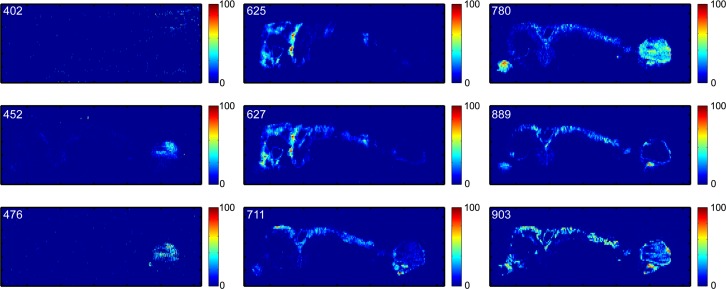
Extracted negative ion DESI-MS images for the nine metabolites in the ET DKO/control serum discriminatory panel not included in the fourth NMF component spectrum.

To further elucidate the relationship between relative metabolite abundance in tissue compared to relative changes detected in serum, extracted ion images for metabolites in the best serum discriminatory panel which were not present in the fourth NMF component were also investigated (Fig 6**).** The feature detected at *m/z* 402.7997 (unassigned metabolite in serum) showed no spatial correlation with any region of the tissue in the extracted ion image. Features detected at *m/z* 452.2783 (LPE 16:0) and 476.2772 (LPE 18:2) both exhibited elevated abundance within the blood-filled cyst region, however, only LPE 18:2 was elevated in DKO mice sera compared to control samples. Features detected at *m/z* 625.4240 (C_42_H_58_O_4_) and 627.5034 (DG 37:5) exhibited very irregular, yet almost identical extracted ion images. This could suggest that the major contributing species to each of these two extracted ion images may be biologically or chemically related. There are two possible explanations for these imaging results: (i) these extracted ion images may be representing chemical species other than C_42_H_58_O_4_ and DG(37:5), or (ii) that the tentative metabolite identification reported for these metabolites in serum could be revised–a possibility as these species were not confirmed by chromatographic comparison to standards. The feature detected at *m/z* 711.3057 exhibited a very unique extracted ion image, showing relatively high abundance in the tumor tissue but not in the blood-filled cyst or the healthy ovary. This feature also remained structurally unidentified in the serum analysis but the selective localization in the tumor tissue suggests it may represent an important biochemical marker worthy of future identification efforts. The feature detected at *m/z* 780.5530, tentatively identified from the serum analysis as PE(39:4), showed relatively high abundance in the healthy ovary and the tumor/cyst regions. This spatial distribution was comparable with other PE lipids detected (*m/z* 716.5247, 722.5070, 746.5053, 766.5324, and 794.5395), except this particular PE species shows a much higher relative abundance in the blood-filled cyst region. Although this metabolite was detected at only slightly elevated levels in DKO mice sera compared to controls (+0.061 log_2_ fold change), the extracted ion image gave a clear indication that this metabolite was associated with the ovary biochemistry and elevated with tumor progression. The feature detected at *m/z* 889.7234 exhibited a spatial distribution similar to some prominent membrane phospholipids, *e*.*g*., PI(38:4), PE(38:4), and PS(36:1). However, this metabolite was tentatively identified as the [M-H]^-^ ion of triacylglycerol TG(55:7) in serum, so further structural investigations may be warranted for this species. The ninth metabolic feature reported in the serum panel and detected by DESI-MS at *m/z* 903.6231 showed a slight abundance increase within parts of the tumor tissue, but it was also present throughout the entire tissue section, making it difficult to draw any significant biological conclusions.

## Conclusion

Applying NMF and DESI-MSI to the reproductive system of a DKO mouse model revealed several metabolites present in tumor tissue and the surrounding blood-filled cyst at elevated levels relative to adjacent healthy tissue. Three ceramide species, Cer(d34:1), Cer(d42:2), and Cer(d42:1) were among the most abundant ions detected, being identified as active metabolites involved in protecting cancerous cells from the host immune system. Eight of the most abundant metabolites present in the selected NMF component, including several lipids and small metabolites, were also detected in sera and used to discriminate ET DKO mice from controls with 88% accuracy. Furthermore, DESI-MSI data was able to link metabolites identified as discriminatory features in a previous serum metabolomic study with their spatial distribution within the tissue, providing support for their biological relevance.

Future work will involve combining both negative- and positive-ion DESI-MS data and expanding to higher spatial resolution MSI methods such as MALDI and secondary-ion mass spectrometry to obtain a more complete picture of molecular changes related to early-stage ovarian cancer progression. These additional ionization techniques will also provide wider metabolome coverage and provide complementary information to that obtained by conventional ESI-MS analyses. Moreover, these approaches could allow us to investigate stage-specific changes in metabolite abundances during the progression of ovarian cancer.

## Supporting Information

S1 Fig The resulting NMF component images and corresponding component spectra from splitting the DESI-MS negative-ion mode dataset into three NMF components. (TIF)

S2 Fig The resulting NMF component images and corresponding component spectra from splitting the DESI-MS negative-ion mode dataset into four NMF components. (TIF)

S3 Fig The resulting K-Means clustering images and corresponding spectra for the DESI-MS negative-ion mode dataset where k = 5. (TIF)
