# Supplementary figures and images for: Whole Reproductive System Non-Negative Matrix Factorization Mass Spectrometry Imaging of an Early-Stage Ovarian Cancer Mouse Model
Source: PLoS One. 2016 May 9;11(5):e0154837. doi: 10.1371/journal.pone.0154837 (PMC4861325; doi:10.1371/journal.pone.0154837)

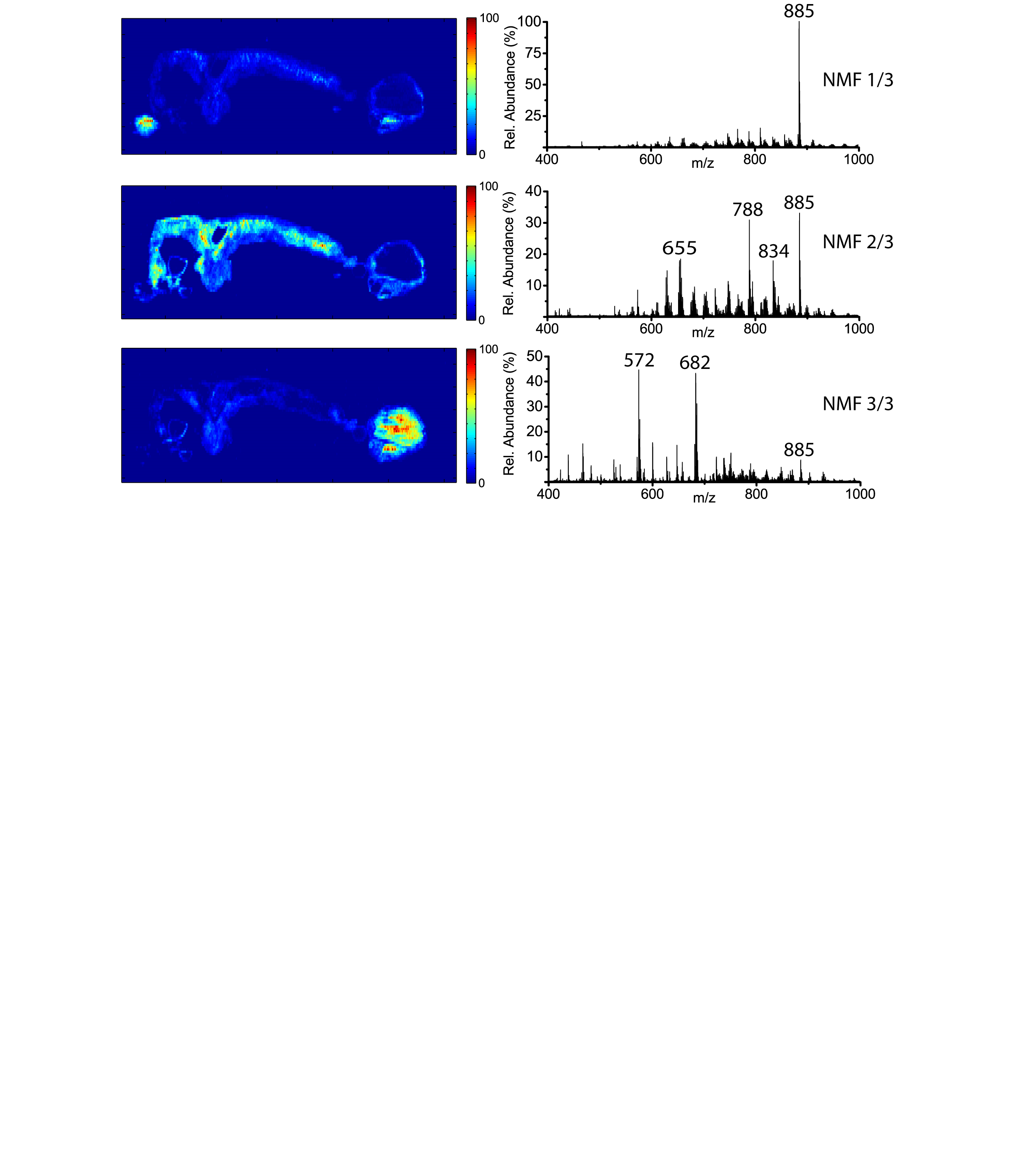

Supplement: S1 Fig — (TIF) [file pone.0154837.s001.tif]

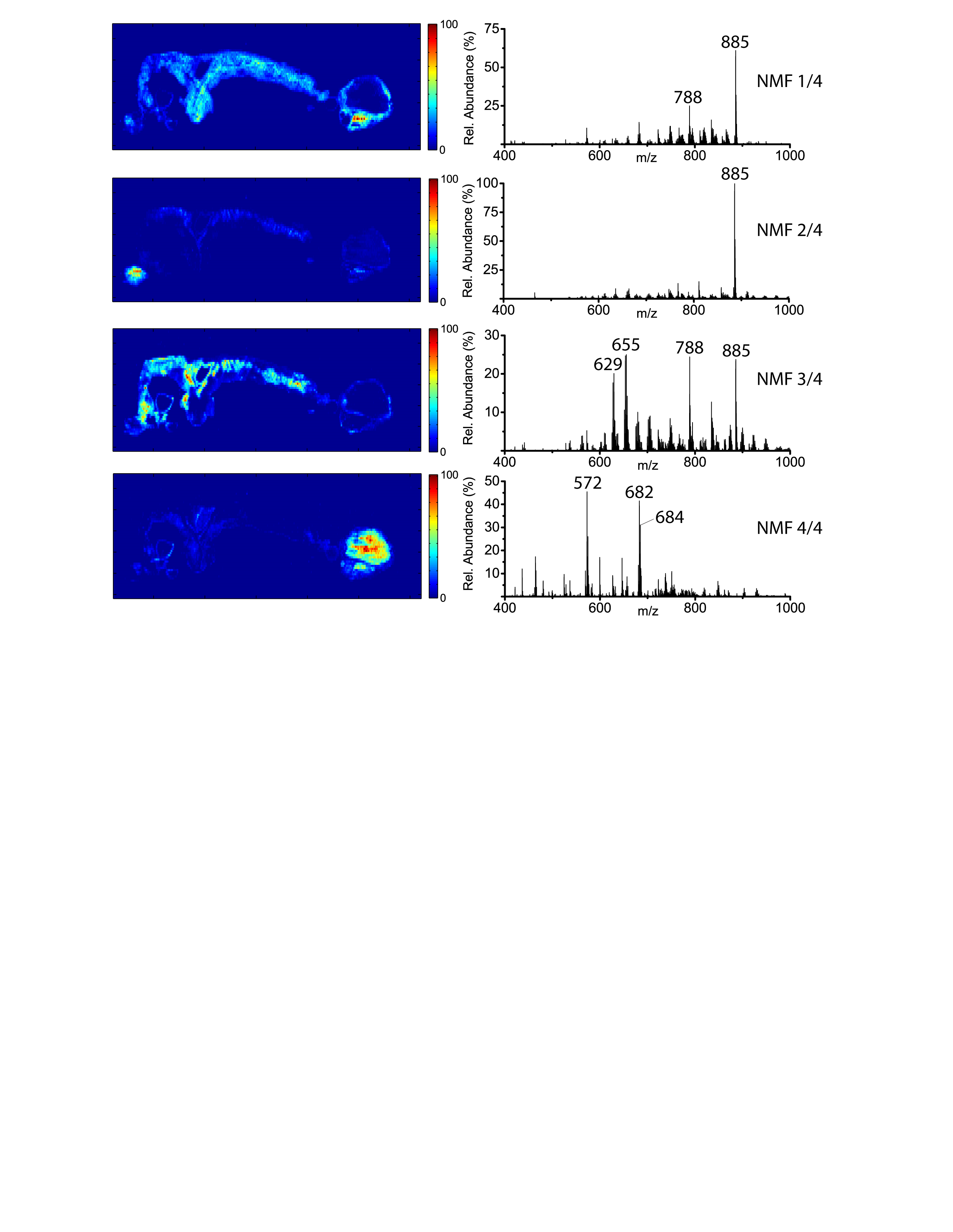

Supplement: S2 Fig — (TIF) [file pone.0154837.s002.tif]

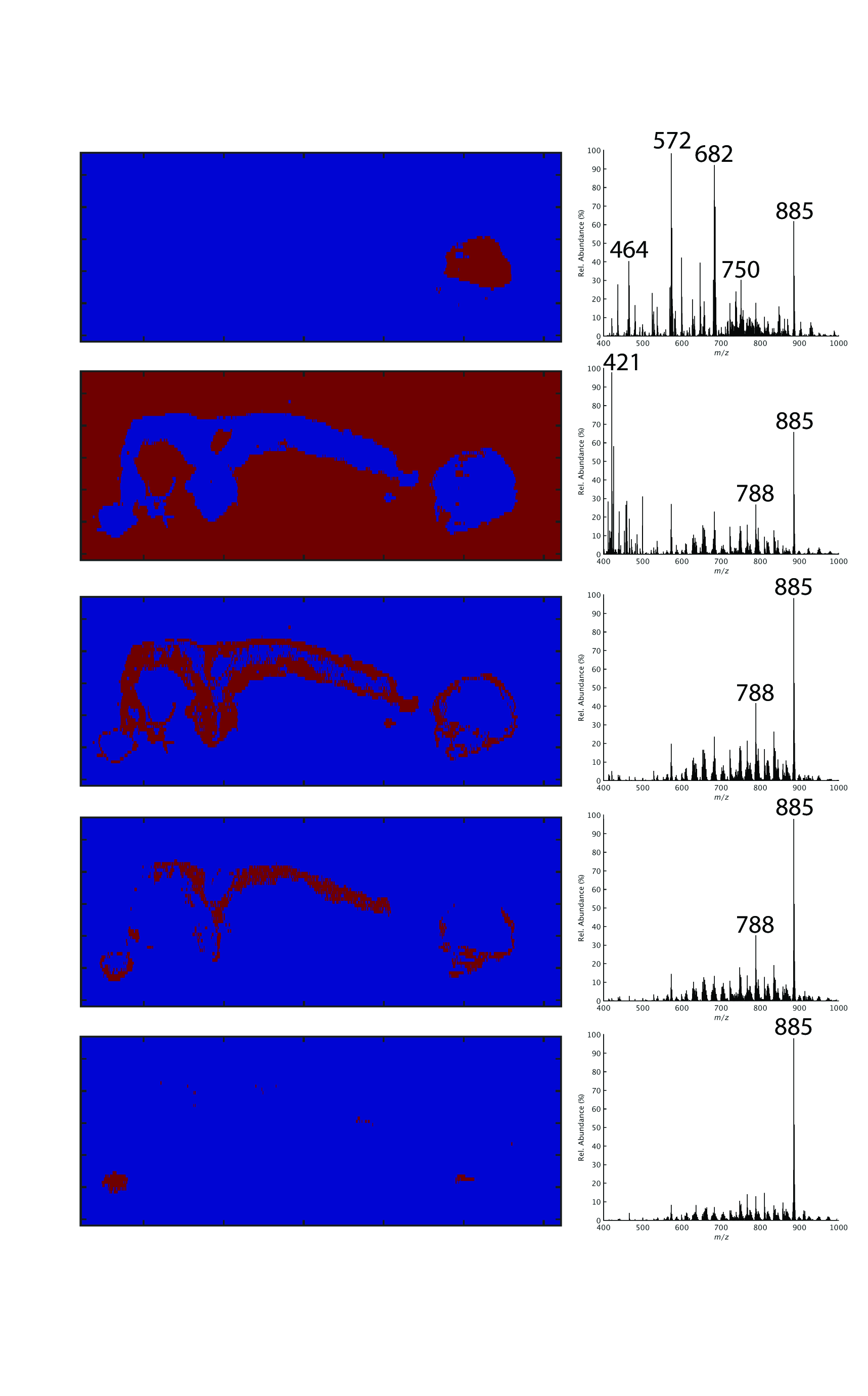

Supplement: S3 Fig — (TIF) [file pone.0154837.s003.tif]
